# Rib fracture management: A review of surgical stabilization, regional analgesia, and intercostal nerve cryoablation

**DOI:** 10.1016/j.sipas.2022.100089

**Published:** 2022-05-16

**Authors:** Stephen Stopenski, Jana Binkley, Sebastian D. Schubl, Zachary M. Bauman

**Affiliations:** a Division of Trauma, Department of Surgery, University of California at Irvine Medical Center, Orange, California, USA; b Division of Trauma, Emergency General Surgery and Critical Care Surgery, Department of Surgery, University of Nebraska Medical Center, Omaha, Nebraska, USA

**Keywords:** Rib fractures, Surgical stabilization of rib fractures, Regional analgesia, Intercostal nerve cryoablation, Pain control

## Abstract

Rib fractures still remain a common problem in blunt thoracic trauma, often resulting significant acute and/or chronic morbidity and mortality. The management of rib fractures has improved over the past two decades, resulting in overall improved patient outcomes. With advances in surgical stabilization of rib fractures (SSRF), improvements in regional analgesia, and the introduction of intercostal nerve cryoablation, patient outcomes from rib fractures have improved significantly over the past several years. This article explores the indications and technique for SSRF as well as discusses additional therapeutic modalities for rib fractures through regional anesthesia and intercostal nerve cryoablation.

## Highlights

• Rib fractures (RF) are common and result in significant morbidity and mortality.
• Surgical stabilization of rib fractures (SSRF) has shown to improve outcomes.
• SSRF indications include flail chest, ≥ 3 severely displaced RF, and the elderly.
• New regional analgesia has improved outcomes and decreased narcotic requirements.
• Intercostal nerve cryoablation is safe and effective pain reducing adjunct to SSRF.

## Background

Rib fractures occur in approximately 10% of all traumatic injury victims [1]. As the number of fractures increase, there is an exponential increase in significant pulmonary-related morbidity and mortality. Almost 1/3 of all chest wall trauma survivors will require prolonged rehabilitation and have issues with chronic pain [1]. Significant progress has been made in the management and treatment of rib fractures over the last few decades, both in the operative and nonoperative management of these patients. Non-operative management, which includes pain control, pulmonary hygiene, and immediate mobilization, has become the mainstay of rib fracture management protocols throughout the world [1]. Epidurals and advancements in local regional anesthesia have also emerged as various pain management options for patients with rib fractures with the caveat they provide only short-term relief. Over the last two decades, however, surgical stabilization of rib fractures (SSRF) has become a pivotal added therapy for these patients [2].

SSRF was first introduced as a successful and safe surgical option for rib fractures in Europe in the 1950’s, specifically as treatment for flail chest injuries [3,4]. It was found when you applied the basic orthopedic principles of reduction and fixation to rib fractures, restoring chest wall stability helped mitigate pain and evade complications from respiratory failure, decreasing medical costs and greatly improving functional outcomes [4,5]. Despite these known benefits and over 50 years of clinical practice, SSRF had remained an underutilized procedure due to lack in technological advancement until the last two decades [5,6]. Traditional SSRF with a large thoracotomy incision was considered a controversial approach due to extensive muscle injury and multiple complications [6]. There was a need for plates and intramedullary fixation devices specifically designed for a rib and its fracture patterns as in the past, surgeons were utilizing whatever bone fixation devices they had available. One of the first rib fixation plate designs, although standardized specifically for rib, was unfortunately linked to high screw pull-out rates and persistent discomfort for the patient, requiring removal in up to 10-15% of patients [6,7]. However, with the evolution of preoperative planning and evolving 3-dimensional computed tomography (CT) reconstruction technology, new rib fixation materials have been developed allowing for individual rib shaping and more minimally invasive surgical techniques [7], [8], [9]. This has resulted in relatively low complication rates, with less than 3% hardware failure rates, and great improvements in overall patient outcomes [7], [8], [9], [10], [11]. Because of these advancements, the utilization of SSRF and literature surrounding this procedure has increased significantly over the past decade [2]. In addition, intercostal nerve cryoablation has emerged as a safe and effective adjunct to SSRF, providing additional rib fracture pain relief for a period of time extending well beyond the patient's hospitalization [5,6]

Although SSRF (with the potential intercostal nerve cryoablation adjunct) is now considered a valid treatment option to limit chest wall-related morbidity, uncertainty still exists as to the group of patients who would benefit from this procedure the most [11]. Flail chest (two fractures on three or more consecutive ribs) patients should be given very strong consideration for SSRF due to the significantly improved outcomes in the literature including decreased mortality, decreased ventilator days, shorter intensive care unit (ICU) and hospital length of stay (LOS), decreased incidence of pneumonia, and decreased need for tracheostomy [12,13]. The role of SSRF in non-flail chest injuries and patients with pulmonary contusions is still somewhat ill-defined in current literature but is becoming more defined as research on this topic continues to increase rapidly. The remainder of this paper will discuss the indications/contraindications for SSRF based on current literature. Furthermore, it will explore the basics of the SSRF technique from both a muscle-sparing open and thoracoscopic approach. Lastly, we will provide an overview of other analgesic adjuncts that can be utilized in the management of rib fracture pain.

## Indications/Contraindications

Nonoperative management, with multimodal pain control and aggressive pulmonary hygiene, is the most common treatment for traumatic rib fractures. However, pain and chest wall instability affect pulmonary function resulting in infections, chronic pain, and long-term disability [14]. Recently, SSRF has been shown to be a cost-effective approach in select patients [15]. The indications for SSRF have evolved and expanded with commercially available systems and favorable prospective trials [16]. To promote consensus among surgeons, The Chest Wall Injury Society (CWIS) is devoted to improving thoracic trauma care and promoting optimal treatment guidelines [17]. Although operative fixation has gained in popularity, the relative number of patients receiving SSRF remains small [1,18]. Selecting candidates for SSRF depends on hospital and surgeon experience. In this section, we outline the current literature to help delineate which patients likely benefit from surgery.

### Flail Chest

The most widely studied indication for SSRF is flail chest or chest wall instability. Although definitions vary, this includes flail chest (fracture of three or more sequential ribs at multiple sites) [19], paradoxical chest motion on exam, and the presence of three consecutive bi-cortically displaced rib fractures [20,21]. Chest wall instability causes ineffective pulmonary mechanisms leading to respiratory failure, mechanical ventilation, and prolonged ICU stays. It is further associated with long-term morbidity such as chronic pain, deformity, and respiratory compromise [22,23]. Even when patients can avoid mechanical ventilation, they still have associated chronic pain and deformity leading to poor quality of life and inability to work [24].

To date, there are four published randomized controlled trials comparing SSRF to optimal medical management in patients with flail chest [25], [26], [27], [28]. All four trials indicate a decrease duration of mechanical ventilation and shorter ICU LOS with surgery. These studies, along with meta-analysis encompassing additional retrospective and prospective studies, overwhelming support SSRF to improve pulmonary outcomes [29], [30], [31], [32], [33]. A review of 22 studies with 988 patients by the Eastern Association for the Surgery of Trauma found SSRF afforded lower mortality, shorter duration of mechanical ventilation, shorter hospital and ICU LOS, as well as lower incidence of pneumonia and need for tracheostomy [30]. A retrospective review of the National Trauma Data Bank including more than 600,000 patients [34] and a recent meta-analysis [33] reiterated a mortality benefit with SSRF in this population. Given the evidence, chest wall instability due to flail chest, in both intubated and non-intubated patients, is an indication for surgery.

### Non-Flail Segment

In half of all rib fracture cases there are three or more consecutive broken ribs, which can result in thoracic deformity, pain, and altered respiratory mechanics [35,36]. Outcomes of patients with severe rib fractures (but without flail chest) have been reported but are typically analyzed together with flail chest patients. In these combined studies, SSRF is associated with better pulmonary outcomes in patients with impending signs of respiratory compromise [20,[37], [38], [39]]. A prospective multicenter study conducted by CWIS focused on whether surgical stabilization in non-flail patients with 3 or more ipsilateral severely displaced (≥ 50% of the rib diameter) fractures was beneficial. They found SSRF was associated with improved pain scores at 2 weeks, fewer pleural complications (e.g. retained hemothorax), and improved respiratory disability-related quality of life. There was no difference in LOS, readmission, pneumonia, or pulmonary function tests. Although the benefits were moderate, their results support the role of SSRF in multiple non-flail displaced rib fractures who have concordant pulmonary derangements such as; respiratory rate ≥20, incentive spirometry volume ≤ 50% predicted, numerical pain score > 5/10, and poor cough [17]. In patients mechanically ventilated, an additional agreed upon indication for SSRF is 3 or more displaced rib fractures with failure to wean ventilator support. This includes patients that failed extubation (reintubated) or persistently fail spontaneous breathing trial after 48 hours.

### Elderly patients

The number of rib fractures correlates with morbidity and mortality, which is particularly true in the elderly [40], [41], [42], [43]. For each additional rib fractured in patients ≥ 65 there is a 27% and 19% associated increase risk of pneumonia and death, respectively [44]. This is likely due to frailty and medical comorbidities making elderly patients less likely to tolerate rib fractures than their younger counterparts. Unfortunately, this creates a paradoxical situation in which elderly patients are less likely to tolerate rib fractures and are simultaneously at higher surgical risk. In patients ≥ 65 years old, multiple studies show that SSRF decreases mortality and respiratory complications (e.g. pneumonia) as well as improve respiratory mechanics [45,46]. Minimally invasive SSRF has been shown to improve post-operative pulmonary function and lead to a more favorable disposition compared to non-operative management [37]. On the other hand, elderly patients have a four-fold increased associated risk of mortality with SSRF compared to younger patients, though the absolute risk of mortality from the operation remains low [8]. Ultimately, these individuals need to be assessed on a case-by-case scenario to weight the risks and benefits of surgery. Vitamin-D and calcium levels should also be obtained and supplemented as necessary. If there is concern for severe osteoporosis, bone mineral density can be assessed through duel-energy x-ray absorptiometry and additional medications can be initiated. However, the authors do not feel osteoporosis should necessarily be a contraindication to SSRF, rather a condition that needs to be optimized to help with bone healing after SSRF.

### TBI Patients

In polytrauma patients, over 15% sustain both traumatic brain injury (TBI) and rib fractures. This dangerous combination is an independent risk factor for pneumonia and in-hospital mortality [47]. TBI exists on a spectrum based on Glasgow Coma Scale (GCS), ranging from severe (GCS ≤ 8) to mild (GCS 13-15). Multiple studies have demonstrated that patients with rib fractures and concurrent closed head injuries have poor outcomes compared to those without head injuries [23,48]. This includes higher rates of mechanical ventilation, chest tube placement, pneumonia, and sepsis. Additionally, patients with head injuries and rib fractures were twice as likely to require tracheostomy and had a mortality rate 3.5 times higher than those without [23].

The benefit of SSRF in moderate and severe TBI is controversial, as these patients have a persistent need for mechanical ventilation that is independent of chest wall physiology. Surgery could contribute to perioperative hypotension or increased intracranial pressure contributing to detrimental secondary brain injury. For these reasons, TBI has traditionally been a contra-indication for SSRF and is excluded from many prospective studies. However, SSRF may still have protective effects against pneumonia. Prins and colleagues found that SSRF in concurrent moderate to severe TBI patients was safe with a low complication rate as well as an associated lower risk of pneumonia and 30-day mortality despite no difference in ventilation-free days [49]. Therefore, TBI should no longer be seen as an absolute contra-indication to surgery and should be considered in select stable moderate and severe TBI patients.

### Additional indications

There are various additional indications where SSRF has been successfully implemented, but supporting data is limited. Patients that “fail” nonoperative management or have rib fractures undergoing thoracic surgery for an additional reason are potential candidates (e.g. “on-the-way-out”). Patients that have delayed or chronic malunion as a result of previous rib fractures may also benefit from SSRF [50]. These patients often remain symptomatic with persistent pain which limits movement months after their injury. That said, delayed repair of non-union and mal-union rib fractures represents an understudied and significantly different patient population from acute fractures and needs to be carefully assessed on a case-by-case basis. Currently, there are no good recommendations in the literature in terms of timing or specific technique for delayed fixation, but the authors do stress the importance of adhering to bone healing principles which often will require the use of a bone graft in these cases. Lastly, acute rib fractures with significant crush injury causing thoracic deformity or with ≥ 30% chest volume loss on axial CT scan have benefited from surgical stabilization [20,51].

### Contraindications

Patients who are hemodynamically unstable and being actively resuscitated should not undergo SSRF. Included in this group are patients with a suspected acute myocardial infarction as they may need antiplatelet/anticoagulation and should avoid additional stress from an invasive procedure [17]. However, a history of shock is not necessarily a contraindication and patients that are intubated and stable may benefit from SSRF as it can reduce sedation and help wean pain medications. As mentioned previously, patients with severe TBI may or may not benefit from SSRF and are at higher risk of morbidity and mortality, therefore require a case-by-case evaluation before performing this procedure. Similarly, unstable spine fractures/spinal cord injuries may preclude safe SSRF and need to be addressed before SSRF is attempted. A high spinal injury may negate benefits of SSRF such as pain control, vent weaning, or avoiding tracheostomy [17]. However, these benefits may still exist for low spinal cord injury with intact chest wall sensation. Lastly, fractures outside ribs 3-10 usually are not fixated given there is no prospective evidence to support the safety and efficacy of doing so, as well as they are not as intimately involved in chest wall mechanics [16,17].

### Technique

Surgical planning starts with a thorough understanding of the fracture pattern, for which CT of the chest is essential. Some authors advocate for three-dimensional reconstruction, but there is no evidence to support the additional expense. The ideal timing of SSRF is uncertain, but continuous respiratory movement can exacerbate pain prompting respiratory decline. Early fixation is associated with decreased operative time and more favorable outcomes [13]. Therefore, fixation is recommended within 72 hours of injury which is in-line with the majority of prospective evidence. Fiber-optic bronchoscopy is often performed in the operating room (OR) to evacuate any mucus plugs and evaluate for large airway injuries. Operative approach can be broadly divided into open and thoracoscopic, which is determined by fracture pattern, experience, and presence of a retained hemothorax.

### Open Technique

Based off imaging, rib fractures can broadly be divided into three zones: anterior, lateral, and posterior. Predominately anterior fractures are approached through an inframammary incision; the patient is positioned supine with arm suspended using a candy cane stirrup or out lateral on arm boards. This allows for bilateral anterior fracture repair without repositioning the patient. This approach should utilize muscle sparing techniques by dividing inferior to the pectoralis muscles and then bluntly dissection the sub-pectoral plane while care is taken to avoid injury to associated nerves. Fractures of the lateral or posterolateral ribs require positioning in the lateral decubitus position. Patients with bilateral fractures are positioned for unilateral SSRF and then either flipped for the contralateral side or have a subsequent operation. The specific incision is tailored to the fracture pattern in a “line of best fit” to the fractures, usually as a vertical axillary or lateral incision. Lateral fractures of three to five levels can be accessed via a longitudinal incision in the mid axillary line. Again, a muscle-sparing technique is recommended by splitting the latissimus dorsi and serratus anterior along their natural muscle fibers. Lastly, a subscapular incision can access the difficult posterior ribs 3 though 7. Occasionally, patients may need to be placed in a prone position for better access and visualization of posterior rib fractures. Fractures at the limit of surgical exposure can be reached with either a right-angle screwdriver system or with the addition of a secondary incision.

Several commercial fixation systems are available, but the basic principles remain constant. Fracture fragments need to be exposed 2-5 cm on either side and the periosteum left on the bone for proper reduction and fixation; any additional exposure is unnecessary and can lead to devascularization. Proper reduction and countertraction can be accomplished by using a variety of clamps packaged with dedicated rib fracture sets, though a penetrating towel clamp is often the most useful instrument to apply gentle upward pressure on the fracture segments. The plates must sit flush with the ribs, and although most systems come pre-contoured, additional bending may be necessary. Fixation can be uni- or bi-cortical depending on the system used. In the case of bicortical fixation, accurate measurement of the rib thickness is critical to ensure use of the correct screw length. Plates should be positioned on the upper two-thirds of the rib to minimize risk of injury to the neurovascular bundle. The adequate number of screws placed to secure the plate differs between systems, so make sure to verify prior to starting starting surgery.

### Thoracoscopic Approach

The natural progression of minimally invasive strategies for SSRF is to a less invasive or completely thoracoscopic approach. Using video assisted thoracoscopic surgery (VATS), rib fractures can be reduced and plated on the inner rib cortex under direct visualization. The potential advantages of a thoracoscopic approach include; minimal trauma to muscles and nerves, improved visualization (specifically posterior and subscapular fractures), and decreased discomfort from palpable or dislodged extra-thoracic plates. Additionally, utilization of a VATS allows for evacuation of retained hemothorax and placement of adjuvant local-regional anesthetics [52]. Patients are positioned in the lateral decubitus position with a bean bag and require double lumen endotracheal intubation for single lung ventilation.

Although considered off-label, conventional extrathoracic plating systems can be utilized for an intrathoracic approach, requiring a total of four incisions for thoracoscopic instruments. The rib fractures are identified intra-thoracic and any retained hemothorax is evacuated. The overlying parietal pleura is incised. The fracture is reduced by using a stab incision in the overlying skin and passing a braided suture around each rib segment with a Carter Thomason CloseSure device (CareFusion, Inc., UK). Next the plate is manually contoured and introduced into the thoracic cavity. The plate is positioned across the fracture line with a Kelly clamp. The surgeon introduces a 90-degree screwdriver to secure the plate to the intrathoracic cortex of the rib. More recently, a commercially available dedicated intrathoracic plating system has become available (RibFix AdvantageTM, Zimmer-Biomet, Jacksonville, FL). This system uses a less invasive approach to fixate a plate to the undersurface of the rib with long wires threaded through the rib and out of the chest via a thoracoport through which the plate can be pulled into the chest. While the system uses VATS to be deployed it still requires some incisions over the fractures for safe and effective deployment. Following SSRF, an intercostal nerve block or cryoablation can be performed. Lastly, a chest tube is placed in the ipsilateral hemithorax.

## Rib Fracture Adjuncts

### Pain Medications

There are several pain medications than can be utilized in the treatment of rib fractures. In fact, studies would advocate the use of multimodal systemic analgesia, which combines opioids and non-opioid medications in an effort to improve pain and decrease opioid consumption [53]. A recent combined guideline from EAST and the Trauma Anesthesiology Society made conditional recommendations in favor of multimodal therapy due to data demonstrating improved analgesia in the setting of known side effects of opioids [53,54]. Additional studies would suggest the use of a multimodal pain regimen to improve pain scores and pulmonary function, advocate quicker return to activities of daily living, and decrease cortisol and adrenocorticotropic hormone levels [[53], [54], [55], [56], [57]]. Given these positive outcomes of multimodal analgesia therapy, the authors of this paper strongly encourage the use of this type of pain regimen, especially for the elderly patient population (≥ 65 years old), as they are the patient population at higher risk of poor outcomes from rib fractures [58].

### Local Regional Analgesia

Regional analgesia techniques are particularly useful in patients with multiple rib fractures, especially those over the age of 65 years [59,60]. Conventional regional techniques used to manage rib fractures include thoracic epidural analgesia (TEA), paravertebral block (PVB), intercostal nerve block (ICNB), serratus anterior block (SAB) and erector spinae block (ESB) [59,61]. Some of these techniques may not be feasible in the presence of anticoagulation, multisystem trauma, or in patients that cannot be properly positioned, so the ability to provide a variety of these techniques for rib fracture patients is extremely efficacious [59,61].

The use of TEA is supported by a number of studies and is perhaps the gold standard for rib fracture regional anesthesia [58,59,[62], [63], [64]]. There have been several studies from both Level 1 and Level 2 centers demonstrating improvements in mortality from the use of TEA in patients with rib fractures [59,63,65]. There have also been some recent retrospective studies and meta-analyses calling into question the efficacy of TEA to reduce pulmonary complications, ICU and hospital LOS, as well as provide mortality benefits [59,[66], [67], [68], [69]]. Despite these questioning studies, a recent EAST guideline has conditionally recommended the use of TEA in traumatic rib fracture patients in view of the low quality of supporting evidence [54,59]. It is important to remember TEA requires both technical competence as well as situational awareness to manage complications such as hypotension, urinary retention, and unintentional blockade [59]. The use of anticoagulation or inability to position a patient may preclude certain patients from TEA [59].

Paravertebral blockade (PVB) has been shown to be as effective as epidurals for pain control in rib fracture patients [59,70,71]. In fact, a recent study demonstrated PVB to be superior than intravenous patient-controlled analgesia when it came to pain control and pulmonary function in this population [59,72]. Given the paravertebral space communicates with the intercostal space laterally and the epidural space medially, a 5-6 dermatome sensory block is possible with a single injection [59,60]. Compared with TEA, a PVB is relatively easy to perform with less side effects given its unilaterality and less effect on the central nervous system, although pneumothorax and local anesthetic toxicity are the major complications to avoid [[59], [60], [61],^73^]. With the use of ultrasound guidance, the analgesic efficacy can be improved substantially [74]. Furthermore, PVBs have the ability to enhance mobility and with recent advances, patients can even be discharged home with some of these various infusion catheters [75].

Recently, ICNBs have been implemented as a means to providing additional pain relief for rib fracture patients. Injecting local anesthetic near the intercostal nerve of all fractured ribs as well as one level above and one level below the group of rib fractures, recent studies have demonstrated improvements in overall respiratory function for these patients [59,60,76]. Although this technique is simple to preform using landmarks or ultrasonography, it has not gained much popularity given the repeated injections further potentiating the risk of pneumothorax [59]. Furthermore, the analgesic effect is short-lived with this procedure.

The SAB was first described by Blanco et al in 2013 and since has gained significant popularity given its efficacy in managing pain associated with rib fractures [77], [78], [79], [80], ease of placement compared to epidurals, and ability to be placed in patients with impaired coagulation [59,77,79,81]. The serratus anterior muscle originates from the anterior surface of the first eight ribs and inserts into the medial boarder of the scapula [59]. Injecting local anesthetic into the myofascial plane surrounding this muscle produces a blockade of the thoracic intercostal nerves T2-T9 resulting in anesthesia to the anterolateral hemithorax[59,81]. Other than neurovascular damage, pneumothorax, and local anesthetic toxicity as potential complications, it is important to remember a SAB is only effective for the anterior two-thirds of the chest wall [59]. Therefore, PVB or ESB should be considered for posterior rib fractures [59].

Erector spinae blocks are directed at the erector spinae myofascial plane, located on the posterior chest wall between the anterior surface of the erector spinae muscle and orientated cephalocaudally to the posterior surface of the spinal transverse process [59,82]. It can provide analgesia to both the anterior and posterior hemithorax and it has been well established for providing adequate pain control for acute rib fractures, pain resulting from thorax or abdominal surgery, and chronic thoracic pain [59]. Lastly, it is a simpler, safer, and less invasive procedure than TEA or PVB, providing extensive truncal analgesia [59]. Given there are no vital structures in the immediate vicinity, the risk of pneumothorax or neurovascular injury is extremely small. Furthermore, it has the advantage of being used in patients with a coagulopathy or on anticoagulation [59].

### Elastomeric Infusion Pumps

Elastomeric infusion pumps (EIP) are catheters placed in the extrathoracic, paraspinous space, designated to create a continuous intercostal nerve block [83]. The EIP has a multiport catheter that infuses medication over a broad area and is connected to a single-use elastomeric reservoir that delivers medication at a defined rate. Although this device has been around for several years serving as a peripheral nerve block elsewhere in the body, its utilization for blocking intercostal nerves after rib fractures has only come into favor over the last few years [83].

A recent study by Truitt et al. examined patients with three or more unilateral rib fractures who underwent placement of an EIP. They found this treatment modality provided significant reduction in numeric pain scores from 9 to 3, which ultimately resulted in decreased narcotic use [83]. Furthermore, they saw significant increases in sustained maximum inspiration from 0.4 L to 1.1 L [83]. Because this catheter is easy to place with minimal risks, it is easy to replicate, providing continuous intercostal nerve blockade, allowing for earlier mobility and overall decreased systemic analgesics. Lastly, patients can have these catheters in place for several days as the elastomeric reservoir can be refilled. Therefore, these patients can be sent home with the EIP in place [83].

### Intercostal Nerve Cryoablation

Cryoablation was first described in the 1970s and relies on the freezing of peripheral nerves in order to produce a prolonged period of analgesia [84], [85], [86]. Although its utilization with postoperative pain control has been around for years, it hasn't been until recently that literature has shown its benefit for rib fracture patients [84]. Intercostal nerve cryoablation (INCA) is a technique performed during surgery in which the intercostal nerve is directly exposed to a probe at -60°Celsius resulting in direct nerve injury known as axonotmesis [84,87,88]. Essentially, the axon and myelin sheath of the nerve are destroyed leaving the endoneural, perineural, and epineural structures intact [84]. Also known as Wallerian degeneration, the patient experiences a numbness sensation distal to the created lesion [84,87,88]. Interestingly, the nerve regenerates at a rate of 3mm/day along these remaining perineural structures, eventually returning to normal sensation as the rib fractures heal [84].

Given this is still a relatively new procedure, the amount of literature on this topic is somewhat limited. The majority of the literature showing INCA's efficacy comes from that of thoracotomy, thoracoscopy, and Nuss procedures [84]. A recent retrospective study by Bauman et al. demonstrated overall shortened hospital LOS, decreased narcotic requirements, as well as a cost benefit when INCA was added to SSRF [84]. A few additional small case series further confirmed this procedure is safe, resulting in decreased pain scores when performed with SSRF [87,89]. Currently, this therapy has mostly been described in the literature being done in concurrence with SSRF, however, a recent case series has emerged showing the benefits and safety of INCA performed percutaneously under ultrasound guidance [90]. Additional studies are required to further investigate the benefits of this therapy, but in its early descriptions with its low side-effect profile, INCA demonstrates significant promise as an adjunct to decreasing the pain from rib fractures and the SSRF surgery itself. It is important to note that long-term data with the use of INCA in rib fractures is still lacking. Although the major complication (traumatic neuroma) has been documented in the literature and usually occurs around 6 weeks post-operatively, there remains a poor understanding of its incidence and need for additional intervention [84]. The authors of this study have performed over 250 cases utilizing INCA and have yet to experience this complication.

## Conclusion

Rib fractures continue to be a challenging problem to manage with mortality rates approaching as high as 50% depending on rib fracture patterns [53,91]. With recent technological advances in the management of chest wall injury, SSRF, along with several other pain adjuncts, have proven safe and advantageous for improving the morbidity and mortality associated with rib fractures. As SSRF continues to gain popularity as a superior treatment modality for rib fractures, it will be important to ensure patient selection is appropriate and surgeons are adequately equipped with the necessary skills to perform this procedure therefore providing its beneficial outcomes.

## Declaration of Competing Interests

Dr. Stephen Stopenski, MD and Dr. Jana Binkley, MD – No financial interests/personal relationships which may be considered as potential competing interests. Dr. Sebastian Schubl is a paid education consultant for Zimmer-Biomet. Dr. Zachary Bauman is a paid educational consultant for Zimmer-Biomet, KLS-Martin, and AtriCure. None of the other authors have any relationships to disclose.
